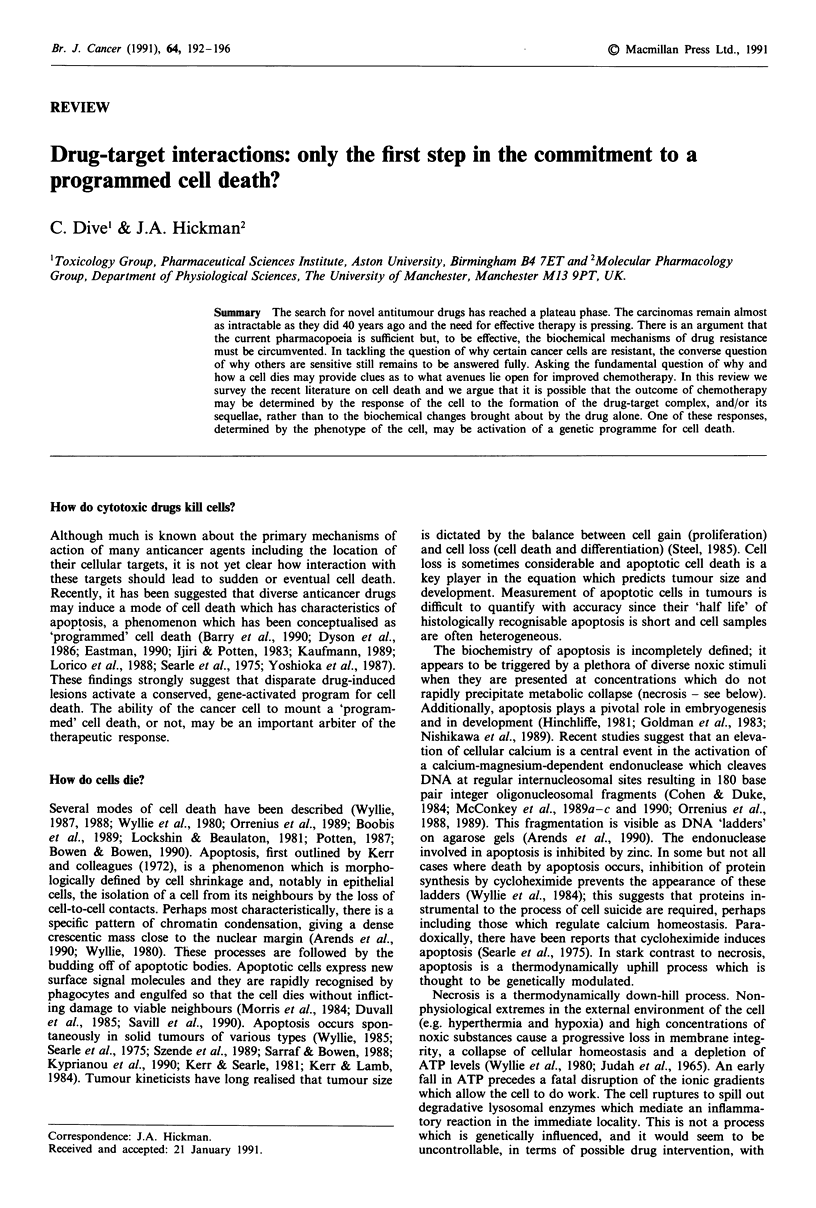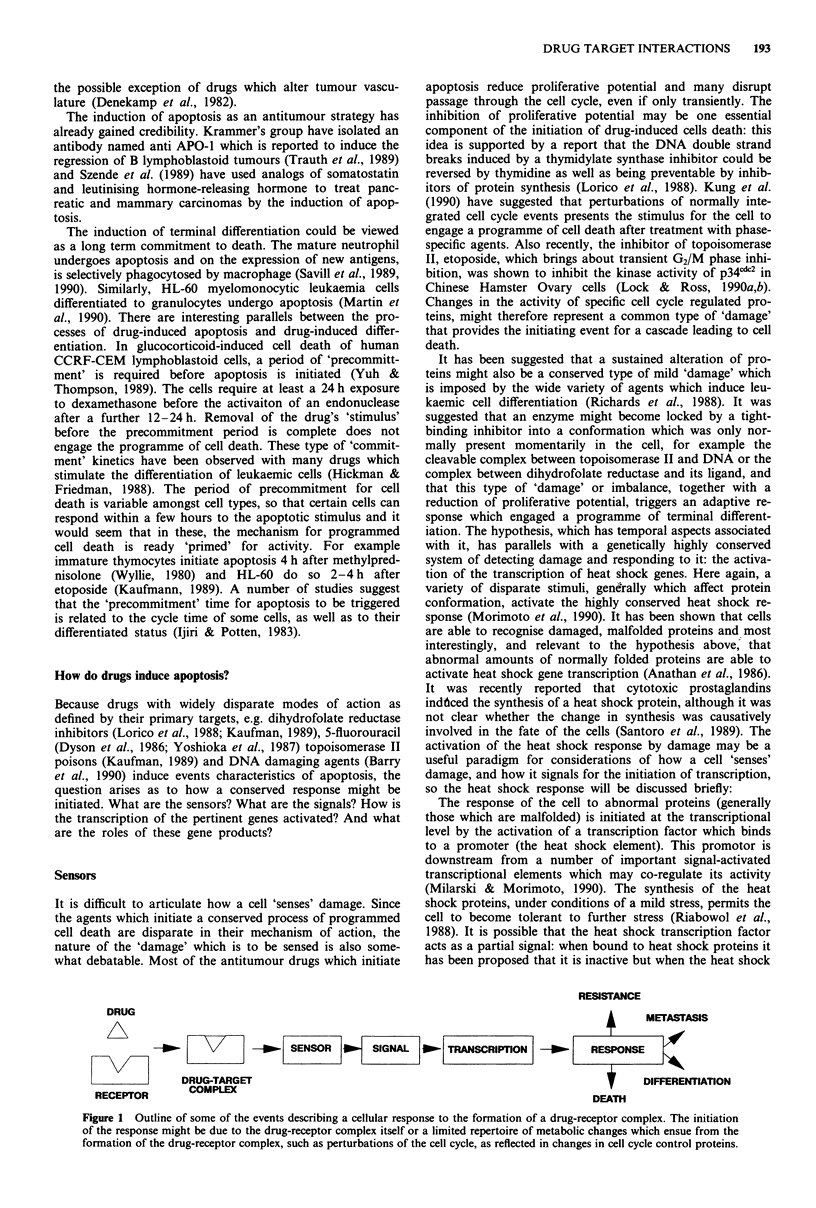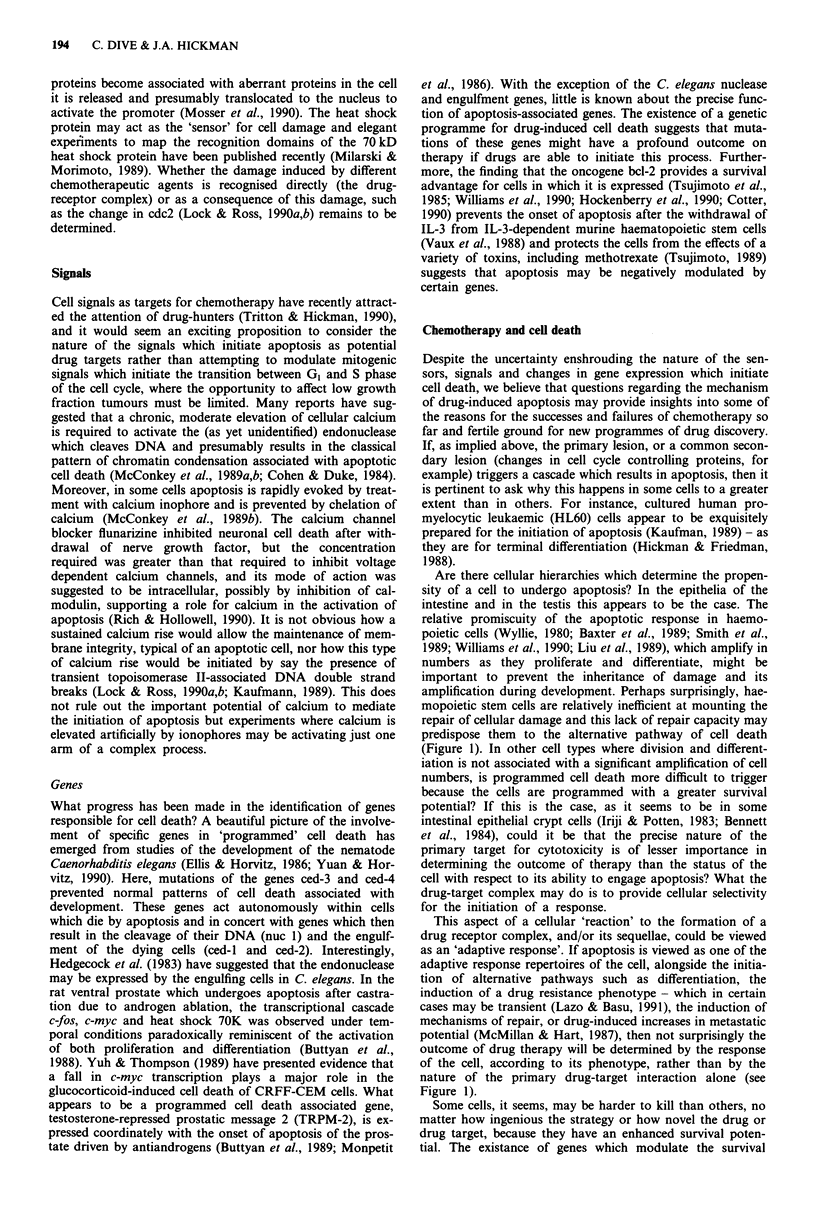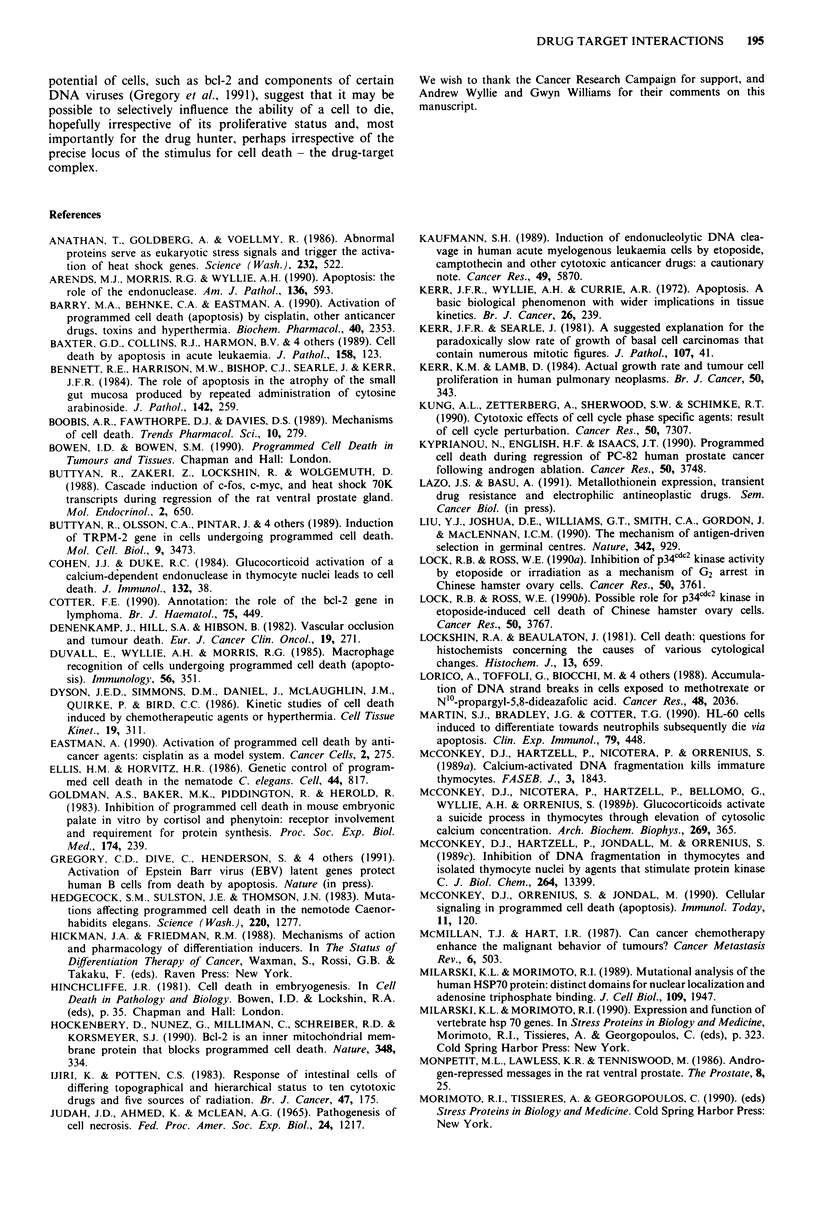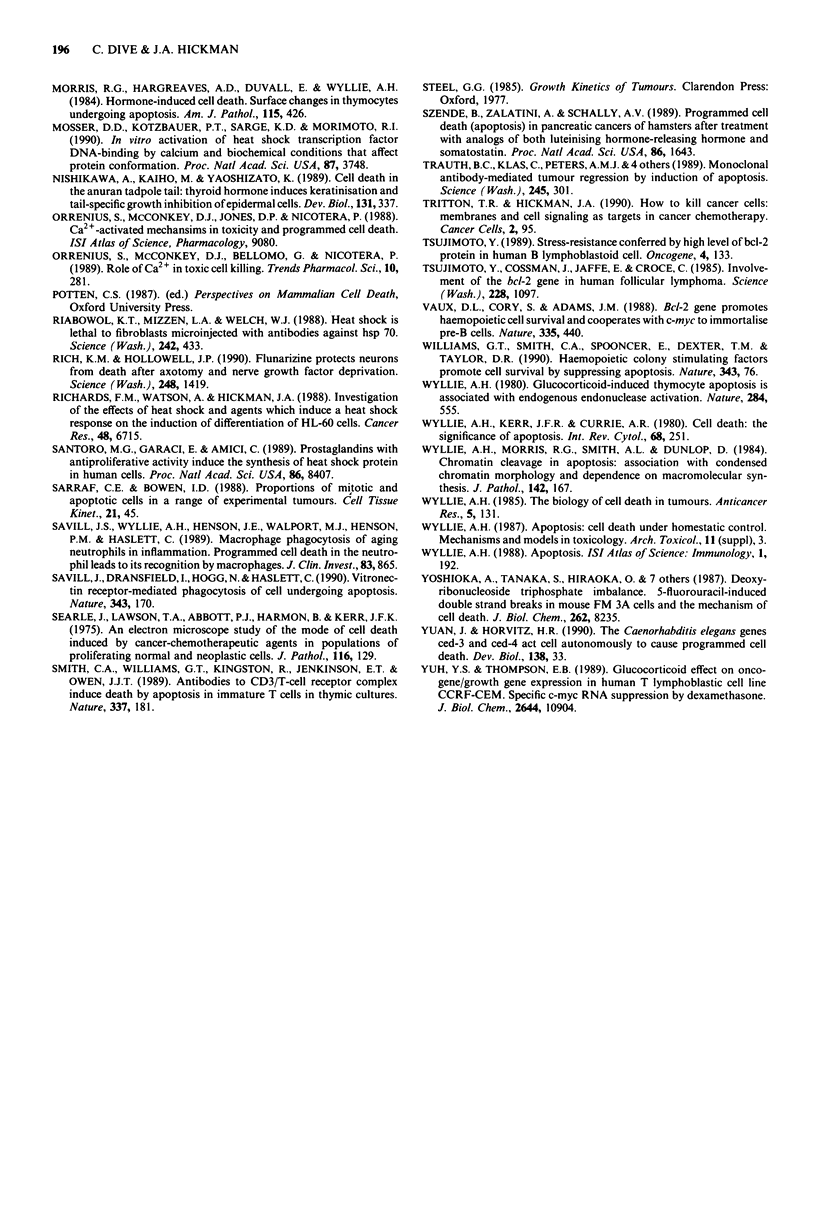# Drug-target interactions: only the first step in the commitment to a programmed cell death?

**DOI:** 10.1038/bjc.1991.269

**Published:** 1991-07

**Authors:** C. Dive, J. A. Hickman

**Affiliations:** Toxicology Group, Pharmaceutical Sciences Institute, Aston University, Birmingham, UK.

## Abstract

The search for novel antitumour drugs has reached a plateau phase. The carcinomas remain almost as intractable as they did 40 years ago and the need for effective therapy is pressing. There is an argument that the current pharmacopoeia is sufficient but, to be effective, the biochemical mechanisms of drug resistance must be circumvented. In tackling the question of why certain cancer cells are resistant, the converse question of why others are sensitive still remains to be answered fully. Asking the fundamental question of why and how a cell dies may provide clues as to what avenues lie open for improved chemotherapy. In this review we survey the recent literature on cell death and we argue that it is possible that the outcome of chemotherapy may be determined by the response of the cell to the formation of the drug-target complex, and/or its sequellae, rather than to the biochemical changes brought about by the drug alone. One of these responses, determined by the phenotype of the cell, may be activation of a genetic programme for cell death.


Br. J. Cancer (1991), 64, 192-196 © Macmillan Press Ltd., 1991

REVIEW

### Drug-target interactions: only the first step in the commitment to a programmed cell death?

C. Dive[1] & J.A. Hickman[2]

[1]Toxicology Group, Pharmaceutical Sciences Institute, Aston University, Birmingham B4 7ET and [2]Molecular Pharmacology Group, Department of Physiological Sciences, The University of Manchester, Manchester M13 9PT, UK.

**Summary** The search for novel antitumour drugs has reached a plateau phase. The carcinomas remain almost as intractable as they did 40 years ago and the need for effective therapy is pressing. There is an argument that the current pharmacopoeia is sufficient but, to be effective, the biochemical mechanisms of drug resistance must be circumvented. In tackling the question of why certain cancer cells are resistant, the converse question of why others are sensitive still remains to be answered fully. Asking the fundamental question of why and how a cell dies may provide clues as to what avenues lie open for improved chemotherapy. In this review we survey the recent literature on cell death and we argue that it is possible that the outcome of chemotherapy may be determined by the response of the cell to the formation of the drug-target complex, and/or its sequellae, rather than to the biochemical changes brought about by the drug alone. One of these responses, determined by the phenotype of the cell, may be activation of a genetic programme for cell death.

### How do cytotoxic drugs kill cells?

Although much is known about the primary mechanisms of action of many anticancer agents including the location of their cellular targets, it is not yet clear how interaction with these targets should lead to sudden or eventual cell death. Recently, it has been suggested that diverse anticancer drugs may induce a mode of cell death which has characteristics of apoptosis, a phenomenon which has been conceptualised as 'programmed' cell death (Barry *et al.*, 1990; Dyson *et al.*, 1986; Eastman, 1990; Ijiri & Potten, 1983; Kaufmann, 1989; Lorico *et al.*, 1988; Searle *et al.*, 1975; Yoshioka *et al.*, 1987). These findings strongly suggest that disparate drug-induced lesions activate a conserved, gene-activated program for cell death. The ability of the cancer cell to mount a 'programmed' cell death, or not, may be an important arbiter of the therapeutic response.

### How do cells die?

Several modes of cell death have been described (Wyllie, 1987, 1988; Wyllie *et al.*, 1980; Orrenius *et al.*, 1989; Boobis *et al.*, 1989; Lockshin & Beaulaton, 1981; Potten, 1987; Bowen & Bowen, 1990). Apoptosis, first outlined by Kerr and colleagues (1972), is a phenomenon which is morphologically defined by cell shrinkage and, notably in epithelial cells, the isolation of a cell from its neighbours by the loss of cell-to-cell contacts. Perhaps most characteristically, there is a specific pattern of chromatin condensation, giving a dense crescentic mass close to the nuclear margin (Arends *et al.*, 1990; Wyllie, 1980). These processes are followed by the budding off of apoptotic bodies. Apoptotic cells express new surface signal molecules and they are rapidly recognised by phagocytes and engulfed so that the cell dies without inflicting damage to viable neighbours (Morris *et al.*, 1984; Duvall *et al.*, 1985; Savill *et al.*, 1990). Apoptosis occurs spontaneously in solid tumours of various types (Wyllie, 1985; Searle *et al.*, 1975; Szende *et al.*, 1989; Sarraf & Bowen, 1988; Kyprianou *et al.*, 1990; Kerr & Searle, 1981; Kerr & Lamb, 1984). Tumour kineticists have long realised that tumour size is dictated by the balance between cell gain (proliferation) and cell loss (cell death and differentiation) (Steel, 1985). Cell loss is sometimes considerable and apoptotic cell death is a key player in the equation which predicts tumour size and development. Measurement of apoptotic cells in tumours is difficult to quantify with accuracy since their 'half life' of histologically recognisable apoptosis is short and cell samples are often heterogeneous.

The biochemistry of apoptosis is incompletely defined; it appears to be triggered by a plethora of diverse noxic stimuli when they are presented at concentrations which do not rapidly precipitate metabolic collapse (necrosis – see below). Additionally, apoptosis plays a pivotal role in embryogenesis and in development (Hinchliffe, 1981; Goldman *et al.*, 1983; Nishikawa *et al.*, 1989). Recent studies suggest that an elevation of cellular calcium is a central event in the activation of a calcium-magnesium-dependent endonuclease which cleaves DNA at regular internucleosomal sites resulting in 180 base pair integer oligonucleosomal fragments (Cohen & Duke, 1984; McConkey *et al.*, 1989*a–c* and 1990; Orrenius *et al.*, 1988, 1989). This fragmentation is visible as DNA 'ladders' on agarose gels (Arends *et al.*, 1990). The endonuclease involved in apoptosis is inhibited by zinc. In some but not all cases where death by apoptosis occurs, inhibition of protein synthesis by cycloheximide prevents the appearance of these ladders (Wyllie *et al.*, 1984); this suggests that proteins instrumental to the process of cell suicide are required, perhaps including those which regulate calcium homeostasis. Paradoxically, there have been reports that cycloheximide induces apoptosis (Searle *et al.*, 1975). In stark contrast to necrosis, apoptosis is a thermodynamically uphill process which is thought to be genetically modulated.

Necrosis is a thermodynamically down-hill process. Nonphysiological extremes in the external environment of the cell (e.g. hyperthermia and hypoxia) and high concentrations of noxic substances cause a progressive loss in membrane integrity, a collapse of cellular homeostasis and a depletion of ATP levels (Wyllie *et al.*, 1980; Judah *et al.*, 1965). An early fall in ATP precedes a fatal disruption of the ionic gradients which allow the cell to do work. The cell ruptures to spill out degradative lysosomal enzymes which mediate an inflammatory reaction in the immediate locality. This is not a process which is genetically influenced, and it would seem to be uncontrollable, in terms of possible drug intervention, with

Correspondence: J.A. Hickman.
Received and accepted: 21 January 1991.

the possible exception of drugs which alter tumour vasculature (Denekamp *et al.*, 1982).

The induction of apoptosis as an antitumour strategy has already gained credibility. Krammer's group have isolated an antibody named anti APO-1 which is reported to induce the regression of B lymphoblastoid tumours (Trauth *et al.*, 1989) and Szende *et al.* (1989) have used analogs of somatostatin and leutinising hormone-releasing hormone to treat pancreatic and mammary carcinomas by the induction of apoptosis.

The induction of terminal differentiation could be viewed as a long term commitment to death. The mature neutrophil undergoes apoptosis and on the expression of new antigens, is selectively phagocytosed by macrophage (Savill *et al.*, 1989, 1990). Similarly, HL-60 myelomonocytic leukaemia cells differentiated to granulocytes undergo apoptosis (Martin *et al.*, 1990). There are interesting parallels between the processes of drug-induced apoptosis and drug-induced differentiation. In glucocorticoid-induced cell death of human CCRF-CEM lymphoblastoid cells, a period of 'precommittment' is required before apoptosis is initiated (Yuh & Thompson, 1989). The cells require at least a 24 h exposure to dexamethasone before the activaiton of an endonuclease after a further 12–24 h. Removal of the drug's 'stimulus' before the precommitment period is complete does not engage the programme of cell death. These type of 'commitment' kinetics have been observed with many drugs which stimulate the differentiation of leukaemic cells (Hickman & Friedman, 1988). The period of precommitment for cell death is variable amongst cell types, so that certain cells can respond within a few hours to the apoptotic stimulus and it would seem that in these, the mechanism for programmed cell death is ready 'primed' for activity. For example immature thymocytes initiate apoptosis 4 h after methylprednisolone (Wyllie, 1980) and HL-60 do so 2–4 h after etoposide (Kaufmann, 1989). A number of studies suggest that the 'precommitment' time for apoptosis to be triggered is related to the cycle time of some cells, as well as to their differentiated status (Ijiri & Potten, 1983).

### How do drugs induce apoptosis?

Because drugs with widely disparate modes of action as defined by their primary targets, e.g. dihydrofolate reductase inhibitors (Lorico *et al.*, 1988; Kaufman, 1989), 5-fluorouracil (Dyson *et al.*, 1986; Yoshioka *et al.*, 1987) topoisomerase II poisons (Kaufman, 1989) and DNA damaging agents (Barry *et al.*, 1990) induce events characteristics of apoptosis, the question arises as to how a conserved response might be initiated. What are the sensors? What are the signals? How is the transcription of the pertinent genes activated? And what are the roles of these gene products?

### Sensors

It is difficult to articulate how a cell 'senses' damage. Since the agents which initiate a conserved process of programmed cell death are disparate in their mechanism of action, the nature of the 'damage' which is to be sensed is also somewhat debatable. Most of the antitumour drugs which initiate apoptosis reduce proliferative potential and many disrupt passage through the cell cycle, even if only transiently. The inhibition of proliferative potential may be one essential component of the initiation of drug-induced cells death: this idea is supported by a report that the DNA double strand breaks induced by a thymidylate synthase inhibitor could be reversed by thymidine as well as being preventable by inhibitors of protein synthesis (Lorico *et al.*, 1988). Kung *et al.* (1990) have suggested that perturbations of normally integrated cell cycle events presents the stimulus for the cell to engage a programme of cell death after treatment with phase-specific agents. Also recently, the inhibitor of topoisomerase II, etoposide, which brings about transient $G_2/M$ phase inhibition, was shown to inhibit the kinase activity of $p34^{cdc2}$ in Chinese Hamster Ovary cells (Lock & Ross, 1990*a*,*b*). Changes in the activity of specific cell cycle regulated proteins, might therefore represent a common type of 'damage' that provides the initiating event for a cascade leading to cell death.

It has been suggested that a sustained alteration of proteins might also be a conserved type of mild 'damage' which is imposed by the wide variety of agents which induce leukaemic cell differentiation (Richards *et al.*, 1988). It was suggested that an enzyme might become locked by a tight-binding inhibitor into a conformation which was only normally present momentarily in the cell, for example the cleavable complex between topoisomerase II and DNA or the complex between dihydrofolate reductase and its ligand, and that this type of 'damage' or imbalance, together with a reduction of proliferative potential, triggers an adaptive response which engaged a programme of terminal differentiation. The hypothesis, which has temporal aspects associated with it, has parallels with a genetically highly conserved system of detecting damage and responding to it: the activation of the transcription of heat shock genes. Here again, a variety of disparate stimuli, generally which affect protein conformation, activate the highly conserved heat shock response (Morimoto *et al.*, 1990). It has been shown that cells are able to recognise damaged, malfolded proteins and most interestingly, and relevant to the hypothesis above, that abnormal amounts of normally folded proteins are able to activate heat shock gene transcription (Anathan *et al.*, 1986). It was recently reported that cytotoxic prostaglandins induced the synthesis of a heat shock protein, although it was not clear whether the change in synthesis was causatively involved in the fate of the cells (Santoro *et al.*, 1989). The activation of the heat shock response by damage may be a useful paradigm for considerations of how a cell 'senses' damage, and how it signals for the initiation of transcription, so the heat shock response will be discussed briefly:

The response of the cell to abnormal proteins (generally those which are malfolded) is initiated at the transcriptional level by the activation of a transcription factor which binds to a promoter (the heat shock element). This promotor is downstream from a number of important signal-activated transcriptional elements which may co-regulate its activity (Milarski & Morimoto, 1990). The synthesis of the heat shock proteins, under conditions of a mild stress, permits the cell to become tolerant to further stress (Riabowol *et al.*, 1988). It is possible that the heat shock transcription factor acts as a partial signal: when bound to heat shock proteins it has been proposed that it is inactive but when the heat shock

**Figure 1** Outline of some of the events describing a cellular response to the formation of a drug-receptor complex. The initiation of the response might be due to the drug-receptor complex itself or a limited repertoire of metabolic changes which ensue from the formation of the drug-receptor complex, such as perturbations of the cell cycle, as reflected in changes in cell cycle control proteins.

proteins become associated with aberrant proteins in the cell it is released and presumably translocated to the nucleus to activate the promoter (Mosser *et al.*, 1990). The heat shock protein may act as the 'sensor' for cell damage and elegant experiments to map the recognition domains of the 70 kD heat shock protein have been published recently (Milarski & Morimoto, 1989). Whether the damage induced by different chemotherapeutic agents is recognised directly (the drug-receptor complex) or as a consequence of this damage, such as the change in cdc2 (Lock & Ross, 1990*a,b*) remains to be determined.

### Signals

Cell signals as targets for chemotherapy have recently attracted the attention of drug-hunters (Tritton & Hickman, 1990), and it would seem an exciting proposition to consider the nature of the signals which initiate apoptosis as potential drug targets rather than attempting to modulate mitogenic signals which initiate the transition between $G_1$ and S phase of the cell cycle, where the opportunity to affect low growth fraction tumours must be limited. Many reports have suggested that a chronic, moderate elevation of cellular calcium is required to activate the (as yet unidentified) endonuclease which cleaves DNA and presumably results in the classical pattern of chromatin condensation associated with apoptotic cell death (McConkey *et al.*, 1989*a,b*; Cohen & Duke, 1984). Moreover, in some cells apoptosis is rapidly evoked by treatment with calcium inophore and is prevented by chelation of calcium (McConkey *et al.*, 1989*b*). The calcium channel blocker flunarizine inhibited neuronal cell death after withdrawal of nerve growth factor, but the concentration required was greater than that required to inhibit voltage dependent calcium channels, and its mode of action was suggested to be intracellular, possibly by inhibition of calmodulin, supporting a role for calcium in the activation of apoptosis (Rich & Hollowell, 1990). It is not obvious how a sustained calcium rise would allow the maintenance of membrane integrity, typical of an apoptotic cell, nor how this type of calcium rise would be initiated by say the presence of transient topoisomerase II-associated DNA double strand breaks (Lock & Ross, 1990*a,b*; Kaufmann, 1989). This does not rule out the important potential of calcium to mediate the initiation of apoptosis but experiments where calcium is elevated artificially by ionophores may be activating just one arm of a complex process.

### *Genes*

What progress has been made in the identification of genes responsible for cell death? A beautiful picture of the involvement of specific genes in 'programmed' cell death has emerged from studies of the development of the nematode *Caenorhabditis elegans* (Ellis & Horvitz, 1986; Yuan & Horvitz, 1990). Here, mutations of the genes ced-3 and ced-4 prevented normal patterns of cell death associated with development. These genes act autonomously within cells which die by apoptosis and in concert with genes which then result in the cleavage of their DNA (nuc 1) and the engulfment of the dying cells (ced-1 and ced-2). Interestingly, Hedgecock *et al.* (1983) have suggested that the endonuclease may be expressed by the engulfing cells in *C. elegans*. In the rat ventral prostate which undergoes apoptosis after castration due to androgen ablation, the transcriptional cascade *c-fos*, *c-myc* and heat shock 70K was observed under temporal conditions paradoxically reminiscent of the activation of both proliferation and differentiation (Buttyan *et al.*, 1988). Yuh & Thompson (1989) have presented evidence that a fall in *c-myc* transcription plays a major role in the glucocorticoid-induced cell death of CRFF-CEM cells. What appears to be a programmed cell death associated gene, testosterone-repressed prostatic message 2 (TRPM-2), is expressed coordinately with the onset of apoptosis of the prostate driven by antiandrogens (Buttyan *et al.*, 1989; Monpetit *et al.*, 1986). With the exception of the *C. elegans* nuclease and engulfment genes, little is known about the precise function of apoptosis-associated genes. The existence of a genetic programme for drug-induced cell death suggests that mutations of these genes might have a profound outcome on therapy if drugs are able to initiate this process. Furthermore, the finding that the oncogene bcl-2 provides a survival advantage for cells in which it is expressed (Tsujimoto *et al.*, 1985; Williams *et al.*, 1990; Hockenberry *et al.*, 1990; Cotter, 1990) prevents the onset of apoptosis after the withdrawal of IL-3 from IL-3-dependent murine haematopoietic stem cells (Vaux *et al.*, 1988) and protects the cells from the effects of a variety of toxins, including methotrexate (Tsujimoto, 1989) suggests that apoptosis may be negatively modulated by certain genes.

### Chemotherapy and cell death

Despite the uncertainty enshrouding the nature of the sensors, signals and changes in gene expression which initiate cell death, we believe that questions regarding the mechanism of drug-induced apoptosis may provide insights into some of the reasons for the successes and failures of chemotherapy so far and fertile ground for new programmes of drug discovery. If, as implied above, the primary lesion, or a common secondary lesion (changes in cell cycle controlling proteins, for example) triggers a cascade which results in apoptosis, then it is pertinent to ask why this happens in some cells to a greater extent than in others. For instance, cultured human promyelocytic leukaemic (HL60) cells appear to be exquisitely prepared for the initiation of apoptosis (Kaufman, 1989) – as they are for terminal differentiation (Hickman & Friedman, 1988).

Are there cellular hierarchies which determine the propensity of a cell to undergo apoptosis? In the epithelia of the intestine and in the testis this appears to be the case. The relative promiscuity of the apoptotic response in haemopoietic cells (Wyllie, 1980; Baxter *et al.*, 1989; Smith *et al.*, 1989; Williams *et al.*, 1990; Liu *et al.*, 1989), which amplify in numbers as they proliferate and differentiate, might be important to prevent the inheritance of damage and its amplification during development. Perhaps surprisingly, haemopoietic stem cells are relatively inefficient at mounting the repair of cellular damage and this lack of repair capacity may predispose them to the alternative pathway of cell death (Figure 1). In other cell types where division and differentiation is not associated with a significant amplification of cell numbers, is programmed cell death more difficult to trigger because the cells are programmed with a greater survival potential? If this is the case, as it seems to be in some intestinal epithelial crypt cells (Iriji & Potten, 1983; Bennett *et al.*, 1984), could it be that the precise nature of the primary target for cytotoxicity is of lesser importance in determining the outcome of therapy than the status of the cell with respect to its ability to engage apoptosis? What the drug-target complex may do is to provide cellular selectivity for the initiation of a response.

This aspect of a cellular 'reaction' to the formation of a drug receptor complex, and/or its sequellae, could be viewed as an 'adaptive response'. If apoptosis is viewed as one of the adaptive response repertoires of the cell, alongside the initiation of alternative pathways such as differentiation, the induction of a drug resistance phenotype – which in certain cases may be transient (Lazo & Basu, 1991), the induction of mechanisms of repair, or drug-induced increases in metastatic potential (McMillan & Hart, 1987), then not surprisingly the outcome of drug therapy will be determined by the response of the cell, according to its phenotype, rather than by the nature of the primary drug-target interaction alone (see Figure 1).

Some cells, it seems, may be harder to kill than others, no matter how ingenious the strategy or how novel the drug or drug target, because they have an enhanced survival potential. The existance of genes which modulate the survival potential of cells, such as bcl-2 and components of certain DNA viruses (Gregory *et al.*, 1991), suggest that it may be possible to selectively influence the ability of a cell to die, hopefully irrespective of its proliferative status and, most importantly for the drug hunter, perhaps irrespective of the precise locus of the stimulus for cell death – the drug-target complex.

We wish to thank the Cancer Research Campaign for support, and Andrew Wyllie and Gwyn Williams for their comments on this manuscript.

### References

ANATHAN, T., GOLDBERG, A. & VOELLMY, R. (1986). Abnormal proteins serve as eukaryotic stress signals and trigger the activation of heat shock genes. *Science (Wash.)*, **232**, 522.

ARENDS, M.J., MORRIS, R.G. & WYLLIE, A.H. (1990). Apoptosis: the role of the endonuclease. *Am. J. Pathol.*, **136**, 593.

BARRY, M.A., BEHNKE, C.A. & EASTMAN, A. (1990). Activation of programmed cell death (apoptosis) by cisplatin, other anticancer drugs, toxins and hyperthermia. *Biochem. Pharmacol.*, **40**, 2353.

BAXTER, G.D., COLLINS, R.J., HARMON, B.V. & 4 others (1989). Cell death by apoptosis in acute leukaemia. *J. Pathol.*, **158**, 123.

BENNETT, R.E., HARRISON, M.W., BISHOP, C.J., SEARLE, J. & KERR, J.F.R. (1984). The role of apoptosis in the atrophy of the small gut mucosa produced by repeated administration of cytosine arabinoside. *J. Pathol.*, **142**, 259.

BOOBIS, A.R., FAWTHORPE, D.J. & DAVIES, D.S. (1989). Mechanisms of cell death. *Trends Pharmacol. Sci.*, **10**, 279.

BOWEN, I.D. & BOWEN, S.M. (1990). *Programmed Cell Death in Tumours and Tissues*. Chapman and Hall: London.

BUTTYAN, R., ZAKERI, Z., LOCKSHIN, R. & WOLGEMUTH, D. (1988). Cascade induction of c-fos, c-myc, and heat shock 70K transcripts during regression of the rat ventral prostate gland. *Mol. Endocrinol.*, **2**, 650.

BUTTYAN, R., OLSSON, C.A., PINTAR, J. & 4 others (1989). Induction of TRPM-2 gene in cells undergoing programmed cell death. *Mol. Cell. Biol.*, **9**, 3473.

COHEN, J.J. & DUKE, R.C. (1984). Glucocorticoid activation of a calcium-dependent endonuclease in thymocyte nuclei leads to cell death. *J. Immunol.*, **132**, 38.

COTTER, F.E. (1990). Annotation: the role of the bcl-2 gene in lymphoma. *Br. J. Haematol.*, **75**, 449.

DENENKAMP, J., HILL, S.A. & HIBSON, B. (1982). Vascular occlusion and tumour death. *Eur. J. Cancer Clin. Oncol.*, **19**, 271.

DUVALL, E., WYLLIE, A.H. & MORRIS, R.G. (1985). Macrophage recognition of cells undergoing programmed cell death (apoptosis). *Immunology*, **56**, 351.

DYSON, J.E.D., SIMMONS, D.M., DANIEL, J., MCLAUGHLIN, J.M., QUIRKE, P. & BIRD, C.C. (1986). Kinetic studies of cell death induced by chemotherapeutic agents or hyperthermia. *Cell Tissue Kinet.*, **19**, 311.

EASTMAN, A. (1990). Activation of programmed cell death by anticancer agents: cisplatin as a model system. *Cancer Cells*, **2**, 275.

ELLIS, H.M. & HORVITZ, H.R. (1986). Genetic control of programmed cell death in the nematode *C. elegans*. *Cell*, **44**, 817.

GOLDMAN, A.S., BAKER, M.K., PIDDINGTON, R. & HEROLD, R. (1983). Inhibition of programmed cell death in mouse embryonic palate in vitro by cortisol and phenytoin: receptor involvement and requirement for protein synthesis. *Proc. Soc. Exp. Biol. Med.*, **174**, 239.

GREGORY, C.D., DIVE, C., HENDERSON, S. & 4 others (1991). Activation of Epstein Barr virus (EBV) latent genes protect human B cells from death by apoptosis. *Nature* (in press).

HEDGECOCK, S.M., SULSTON, J.E. & THOMSON, J.N. (1983). Mutations affecting programmed cell death in the nemotode Caenorhabidits elegans. *Science (Wash.)*, **220**, 1277.

HICKMAN, J.A. & FRIEDMAN, R.M. (1988). Mechanisms of action and pharmacology of differentiation inducers. In *The Status of Differentiation Therapy of Cancer*, Waxman, S., Rossi, G.B. & Takaku, F. (eds). Raven Press: New York.

HINCHCLIFFE, J.R. (1981). Cell death in embryogenesis. In *Cell Death in Pathology and Biology*. Bowen, I.D. & Lockshin, R.A. (eds), p. 35. Chapman and Hall: London.

HOCKENBERY, D., NUNEZ, G., MILLIMAN, C., SCHREIBER, R.D. & KORSMEYER, S.J. (1990). Bcl-2 is an inner mitochondrial membrane protein that blocks programmed cell death. *Nature*, **348**, 334.

IJIRI, K. & POTTEN, C.S. (1983). Response of intestinal cells of differing topographical and hierarchical status to ten cytotoxic drugs and five sources of radiation. *Br. J. Cancer*, **47**, 175.

JUDAH, J.D., AHMED, K. & MCLEAN, A.G. (1965). Pathogenesis of cell necrosis. *Fed. Proc. Amer. Soc. Exp. Biol.*, **24**, 1217.

KAUFMANN, S.H. (1989). Induction of endonucleolytic DNA cleavage in human acute myelogenous leukaemia cells by etoposide, camptothecin and other cytotoxic anticancer drugs: a cautionary note. *Cancer Res.*, **49**, 5870.

KERR, J.F.R., WYLLIE, A.H. & CURRIE, A.R. (1972). Apoptosis. A basic biological phenomenon with wider implications in tissue kinetics. *Br. J. Cancer*, **26**, 239.

KERR, J.F.R. & SEARLE, J. (1981). A suggested explanation for the paradoxically slow rate of growth of basal cell carcinomas that contain numerous mitotic figures. *J. Pathol.*, **107**, 41.

KERR, K.M. & LAMB, D. (1984). Actual growth rate and tumour cell proliferation in human pulmonary neoplasms. *Br. J. Cancer*, **50**, 343.

KUNG, A.L., ZETTERBERG, A., SHERWOOD, S.W. & SCHIMKE, R.T. (1990). Cytotoxic effects of cell cycle phase specific agents: result of cell cycle perturbation. *Cancer Res.*, **50**, 7307.

KYPRIANOU, N., ENGLISH, H.F. & ISAACS, J.T. (1990). Programmed cell death during regression of PC-82 human prostate cancer following androgen ablation. *Cancer Res.*, **50**, 3748.

LAZO, J.S. & BASU, A. (1991). Metallothionein expression, transient drug resistance and electrophilic antineoplastic drugs. *Sem. Cancer Biol.* (in press).

LIU, Y.J., JOSHUA, D.E., WILLIAMS, G.T., SMITH, C.A., GORDON, J. & MACLENNAN, I.C.M. (1990). The mechanism of antigen-driven selection in germinal centres. *Nature*, **342**, 929.

LOCK, R.B. & ROSS, W.E. (1990*a*). Inhibition of $p34^{cdc2}$ kinase activity by etoposide or irradiation as a mechanism of $G_2$ arrest in Chinese hamster ovary cells. *Cancer Res.*, **50**, 3761.

LOCK, R.B. & ROSS, W.E. (1990*b*). Possible role for $p34^{cdc2}$ kinase in etoposide-induced cell death of Chinese hamster ovary cells. *Cancer Res.*, **50**, 3767.

LOCKSHIN, R.A. & BEAULATON, J. (1981). Cell death: questions for histochemists concerning the causes of various cytological changes. *Histochem. J.*, **13**, 659.

LORICO, A., TOFFOLI, G., BIOCCHI, M. & 4 others (1988). Accumulation of DNA strand breaks in cells exposed to methotrexate or $N^{10}$-propargyl-5,8-dideazafolic acid. *Cancer Res.*, **48**, 2036.

MARTIN, S.J., BRADLEY, J.G. & COTTER, T.G. (1990). HL-60 cells induced to differentiate towards neutrophils subsequently die *via* apoptosis. *Clin. Exp. Immunol.*, **79**, 448.

MCCONKEY, D.J., HARTZELL, P., NICOTERA, P. & ORRENIUS, S. (1989*a*). Calcium-activated DNA fragmentation kills immature thymocytes. *FASEB. J.*, **3**, 1843.

MCCONKEY, D.J., NICOTERA, P., HARTZELL, P., BELLOMO, G., WYLLIE, A.H. & ORRENIUS, S. (1989*b*). Glucocorticoids activate a suicide process in thymocytes through elevation of cytosolic calcium concentration. *Arch. Biochem. Biophys.*, **269**, 365.

MCCONKEY, D.J., HARTZELL, P., JONDALL, M. & ORRENIUS, S. (1989*c*). Inhibition of DNA fragmentation in thymocytes and isolated thymocyte nuclei by agents that stimulate protein kinase C. *J. Biol. Chem.*, **264**, 13399.

MCCONKEY, D.J., ORRENIUS, S. & JONDAL, M. (1990). Cellular signaling in programmed cell death (apoptosis). *Immunol. Today*, **11**, 120.

MCMILLAN, T.J. & HART, I.R. (1987). Can cancer chemotherapy enhance the malignant behavior of tumours? *Cancer Metastasis Rev.*, **6**, 503.

MILARSKI, K.L. & MORIMOTO, R.I. (1989). Mutational analysis of the human HSP70 protein: distinct domains for nuclear localization and adenosine triphosphate binding. *J. Cell Biol.*, **109**, 1947.

MILARSKI, K.L. & MORIMOTO, R.I. (1990). Expression and function of vertebrate hsp 70 genes. In *Stress Proteins in Biology and Medicine*, Morimoto, R.I., Tissieres, A. & Georgopoulos, C. (eds), p. 323. Cold Spring Harbor Press: New York.

MONPETIT, M.L., LAWLESS, K.R. & TENNISWOOD, M. (1986). Androgen-repressed messages in the rat ventral prostate. *The Prostate*, **8**, 25.

MORIMOTO, R.I., TISSIERES, A. & GEORGOPOULOS, C. (1990). (eds) *Stress Proteins in Biology and Medicine*. Cold Spring Harbor Press: New York.

MORRIS, R.G., HARGREAVES, A.D., DUVALL, E. & WYLLIE, A.H. (1984). Hormone-induced cell death. Surface changes in thymocytes undergoing apoptosis. *Am. J. Pathol.*, **115**, 426.

MOSSER, D.D., KOTZBAUER, P.T., SARGE, K.D. & MORIMOTO, R.I. (1990). *In vitro* activation of heat shock transcription factor DNA-binding by calcium and biochemical conditions that affect protein conformation. *Proc. Natl Acad. Sci. USA*, **87**, 3748.

NISHIKAWA, A., KAIHO, M. & YAOSHIZATO, K. (1989). Cell death in the anuran tadpole tail: thyroid hormone induces keratinisation and tail-specific growth inhibition of epidermal cells. *Dev. Biol.*, **131**, 337.

ORRENIUS, S., McCONKEY, D.J., JONES, D.P. & NICOTERA, P. (1988). $Ca^{2+}$-activated mechansims in toxicity and programmed cell death. *ISI Atlas of Science, Pharmacology*, 9080.

ORRENIUS, S., McCONKEY, D.J., BELLOMO, G. & NICOTERA, P. (1989). Role of $Ca^{2+}$ in toxic cell killing. *Trends Pharmacol. Sci.*, **10**, 281.

POTTEN, C.S. (1987). (ed.) *Perspectives on Mammalian Cell Death*, Oxford University Press.

RIABOWOL, K.T., MIZZEN, L.A. & WELCH, W.J. (1988). Heat shock is lethal to fibroblasts microinjected with antibodies against hsp 70. *Science (Wash.)*, **242**, 433.

RICH, K.M. & HOLLOWELL, J.P. (1990). Flunarizine protects neurons from death after axotomy and nerve growth factor deprivation. *Science (Wash.)*, **248**, 1419.

RICHARDS, F.M., WATSON, A. & HICKMAN, J.A. (1988). Investigation of the effects of heat shock and agents which induce a heat shock response on the induction of differentiation of HL-60 cells. *Cancer Res.*, **48**, 6715.

SANTORO, M.G., GARACI, E. & AMICI, C. (1989). Prostaglandins with antiproliferative activity induce the synthesis of heat shock protein in human cells. *Proc. Natl Acad. Sci. USA*, **86**, 8407.

SARRAF, C.E. & BOWEN, I.D. (1988). Proportions of mitotic and apoptotic cells in a range of experimental tumours. *Cell Tissue Kinet.*, **21**, 45.

SAVILL, J.S., WYLLIE, A.H., HENSON, J.E., WALPORT, M.J., HENSON, P.M. & HASLETT, C. (1989). Macrophage phagocytosis of aging neutrophils in inflammation. Programmed cell death in the neutrophil leads to its recognition by macrophages. *J. Clin. Invest.*, **83**, 865.

SAVILL, J., DRANSFIELD, I., HOGG, N. & HASLETT, C. (1990). Vitronectin receptor-mediated phagocytosis of cell undergoing apoptosis. *Nature*, **343**, 170.

SEARLE, J., LAWSON, T.A., ABBOTT, P.J., HARMON, B. & KERR, J.F.K. (1975). An electron microscope study of the mode of cell death induced by cancer-chemotherapeutic agents in populations of proliferating normal and neoplastic cells. *J. Pathol.*, **116**, 129.

SMITH, C.A., WILLIAMS, G.T., KINGSTON, R., JENKINSON, E.T. & OWEN, J.J.T. (1989). Antibodies to CD3/T-cell receptor complex induce death by apoptosis in immature T cells in thymic cultures. *Nature*, **337**, 181.

STEEL, G.G. (1985). *Growth Kinetics of Tumours*. Clarendon Press: Oxford, 1977.

SZENDE, B., ZALATINI, A. & SCHALLY, A.V. (1989). Programmed cell death (apoptosis) in pancreatic cancers of hamsters after treatment with analogs of both luteinising hormone-releasing hormone and somatostatin. *Proc. Natl Acad. Sci. USA*, **86**, 1643.

TRAUTH, B.C., KLAS, C., PETERS, A.M.J. & 4 others (1989). Monoclonal antibody-mediated tumour regression by induction of apoptosis. *Science (Wash.)*, **245**, 301.

TRITTON, T.R. & HICKMAN, J.A. (1990). How to kill cancer cells: membranes and cell signaling as targets in cancer chemotherapy. *Cancer Cells*, **2**, 95.

TSUJIMOTO, Y. (1989). Stress-resistance conferred by high level of bcl-2 protein in human B lymphoblastoid cell. *Oncogene*, **4**, 133.

TSUJIMOTO, Y., COSSMAN, J., JAFFE, E. & CROCE, C. (1985). Involvement of the *bcl*-2 gene in human follicular lymphoma. *Science (Wash.)*, **228**, 1097.

VAUX, D.L., CORY, S. & ADAMS, J.M. (1988). *Bcl*-2 gene promotes haemopoietic cell survival and cooperates with c-*myc* to immortalise pre-B cells. *Nature*, **335**, 440.

WILLIAMS, G.T., SMITH, C.A., SPOONCER, E., DEXTER, T.M. & TAYLOR, D.R. (1990). Haemopoietic colony stimulating factors promote cell survival by suppressing apoptosis. *Nature*, **343**, 76.

WYLLIE, A.H. (1980). Glucocorticoid-induced thymocyte apoptosis is associated with endogenous endonuclease activation. *Nature*, **284**, 555.

WYLLIE, A.H., KERR, J.F.R. & CURRIE, A.R. (1980). Cell death: the significance of apoptosis. *Int. Rev. Cytol.*, **68**, 251.

WYLLIE, A.H., MORRIS, R.G., SMITH, A.L. & DUNLOP, D. (1984). Chromatin cleavage in apoptosis: association with condensed chromatin morphology and dependence on macromolecular synthesis. *J. Pathol.*, **142**, 167.

WYLLIE, A.H. (1985). The biology of cell death in tumours. *Anticancer Res.*, **5**, 131.

WYLLIE, A.H. (1987). Apoptosis: cell death under homestatic control. Mechanisms and models in toxicology. *Arch. Toxicol.*, **11** (suppl), 3.

WYLLIE, A.H. (1988). Apoptosis. *ISI Atlas of Science: Immunology*, **1**, 192.

YOSHIOKA, A., TANAKA, S., HIRAOKA, O. & 7 others (1987). Deoxyribonucleoside triphosphate imbalance. 5-fluorouracil-induced double strand breaks in mouse FM 3A cells and the mechanism of cell death. *J. Biol. Chem.*, **262**, 8235.

YUAN, J. & HORVITZ, H.R. (1990). The *Caenorhabditis elegans* genes ced-3 and ced-4 act cell autonomously to cause programmed cell death. *Dev. Biol.*, **138**, 33.

YUH, Y.S. & THOMPSON, E.B. (1989). Glucocorticoid effect on oncogene/growth gene expression in human T lymphoblastic cell line CCRF-CEM. Specific c-myc RNA suppression by dexamethasone. *J. Biol. Chem.*, **2644**, 10904.